# 5-Formylcytosine landscapes of human preimplantation embryos at single-cell resolution

**DOI:** 10.1371/journal.pbio.3000799

**Published:** 2020-07-30

**Authors:** Yun Gao, Lin Li, Peng Yuan, Fan Zhai, Yixin Ren, Liying Yan, Rong Li, Ying Lian, Xiaohui Zhu, Xinglong Wu, Kehkooi Kee, Lu Wen, Jie Qiao, Fuchou Tang

**Affiliations:** 1 Beijing Advanced Innovation Center for Genomics, Department of Obstetrics and Gynecology, School of Life Sciences, Third Hospital, Peking University, Beijing, China; 2 Biomedical Pioneering Innovaiton Center, Ministry of Education Key Laboratory of Cell Proliferation and Differentiation, Peking University, Beijing, China; 3 Guangdong Provincial Key Laboratory of Proteomics, Department of Pathophysiology, School of Basic Medical Sciences, Southern Medical University, Guangzhou, China; 4 Key Laboratory of Assisted Reproduction, Ministry of Education, Beijing, China; 5 Beijing Key Laboratory of Reproductive Endocrinology and Assisted Reproductive Technology, Beijing, China; 6 Peking-Tsinghua Center for Life Sciences, Peking University, Beijing, China; 7 Academy for Advanced Interdisciplinary Studies, Peking University, Beijing, China; 8 Center for Stem Cell Biology and Regenerative Medicine, Department of Basic Medical Sciences, School of Medicine, Tsinghua University, Beijing, China; Max F Perutz Laboratories Center of Molecular Biology, AUSTRIA

## Abstract

Epigenetic dynamics, such as DNA methylation and chromatin accessibility, have been extensively explored in human preimplantation embryos. However, the active demethylation process during this crucial period remains largely unexplored. In this study, we use single-cell chemical-labeling-enabled C-to-T conversion sequencing (CLEVER-seq) to quantify the DNA 5-formylcytosine (5fC) levels of human preimplantation embryos. We find that 5-formylcytosine phosphate guanine (5fCpG) exhibits genomic element-specific distribution features and is enriched in L1 and endogenous retrovirus-K (ERVK), the subfamilies of repeat elements long interspersed nuclear elements (LINEs) and long terminal repeats (LTRs), respectively. Unlike in mice, paired pronuclei in the same zygote present variable difference of 5fCpG levels, although the male pronuclei experience stronger global demethylation. The nucleosome-occupied regions show a higher 5fCpG level compared with nucleosome-depleted ones, suggesting the role of 5fC in organizing nucleosome position. Collectively, our work offers a valuable resource for ten-eleven translocation protein family (TET)-dependent active demethylation-related study during human early embryonic development.

## Abstract 2

The use of CLEVER-seq to delineate the 5-formylcytosine landscape of human early embryos at single-cell resolution enhances our understanding of TET-dependent active demethylation processes during human preimplantation embryo development.

## Introduction

Epigenetic regulation is crucial for early embryonic development to control the expression of key regulators to complete the reprogramming process [1–4]. The dynamics of DNA methylation and chromatin accessibility have been extensively analyzed in human preimplantation embryos [5–11]. Human early embryos go through waves of global demethylation after fertilization [12]. Active demethylation is mediated by the ten-eleven translocation protein family (TET) to produce a series of oxidized derivatives of 5-methylcytosine (5mC), including 5-hydroxymethylcytosine (5hmC), 5-formylcytosine (5fC), and 5-carboxylcytosine (5caC) [13–17]. As an essential active demethylation status, 5fC can organize the nucleosome position and be occupied by corresponding regulatory proteins [18–20]. Additionally, the production of 5fC in promoters and tissue-specific enhancers is usually associated with the up-regulation of gene expression [18,21,22]. However, the genome-wide 5fC dynamics during human preimplantation development are largely unknown. Here, we performed single-cell chemical-labeling-enabled C-to-T conversion sequencing (CLEVER-seq) to dissect the 5fC landscapes of human early embryos to provide new insights into the potential function of active demethylation in this process.

## Results

### Genome-wide identification of 5fC during human early embryonic development

**Fig 1:**
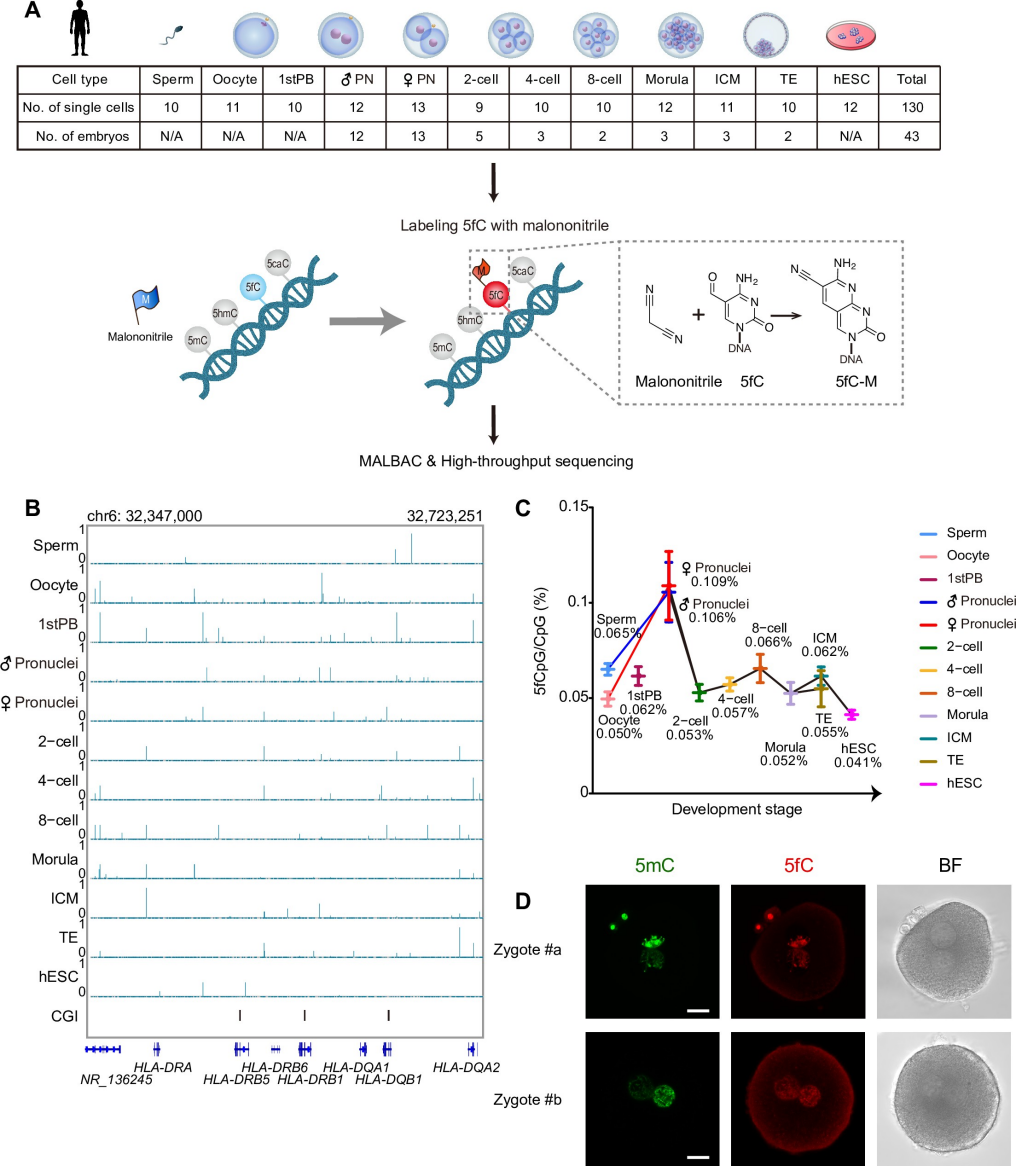
The DNA formylation dynamic of human early embryos and hESC. (A) The flowchart of CLEVER-seq and sample information. The number of sperm cells, oocytes, the first polar bodies, male pronuclei, female pronuclei, blastomeres of two-cell, four-cell, eight-cell, morula, ICM and TE, and hESCs without CNV are listed in the top table, as well as the number of embryos. The sampling time of pronucleus at zygote stage is 16 to 18 h after ICSI. (B) The UCSC browse view showing the 5fCpG sites distribution across the human early embryo developmental stages. (C) The line chart showing 5fCpG level throughout the human early embryonic development. The 5fCpG percentage was calculated by the number of 5fCpG sites divided by the sum of 5fCpG sites and unmodified CpG sites. The center is the mean of 5fCpG level and error bars are SEM. The sample size are listed in Fig 1A. The numerical data is listed in S1 Table. (D) The representative immunostaining image of 5mC and 5fC in human zygotes. The scale bar represents 20 μm. BF, bright field; CGI, CpG island; CLEVER-seq, chemical-labeling-enabled C-to-T conversion sequencing; CNV, copy number variation; CpG, cytosine phosphate guanine; hESC, human embryonic stem cell; ICM, inner cell mass; ICSI, intracytoplasmic sperm injection; MALBAC, multiple annealing- and looping-based amplification cycles; SEM, standard error of the mean; TE, trophectoderm; UCSC, University of California, Santa Cruz; 5fCpG, 5-formylcytosine phosphate guanine; 5mC, 5-methylcytosine.

5fC is a relatively rare DNA modification in the genome, with only 20 to 200 ppm of cytosines [15,16,23–26]. To map 5fC in a single cell’s genome, we applied CLEVER-seq to human gametes, the first polar bodies, human preimplantation embryos at six key developmental stages (zygotes, 2-cell, 4-cell, 8-cell embryos, morulae, and blastocysts) and human embryonic stem cells (hESCs). In total, we obtained 130 euploid individual cells without copy number variations (CNVs) for further analysis (Fig 1A, S1A Fig and S1 Table). In CLEVER-seq, malononitrile is a key chemical that can specifically label 5fC with high efficiency [22]. The synthesized model DNA containing 5fC modifications was spiked into each sample to quantify the efficiency of malononitrile labeling (S2 Table). The average conversion rate was 79.6% in all 130 malononitrile-treated single-cell samples (S1B Fig). With approximately 5× sequencing depth for each malononitrile-treated single-cell sample, the average number of clean reads of each sample was 117.8 million with a 74.6% averaged mapping rate (S1C Fig). On average, 9.0 million, 6.4 million, and 5.0 million unique cytosine phosphate guanine (CpG) sites were covered in each malononitrile-treated sample at ≥1×, ≥3×, and ≥5× coverage, respectively (S1D and S1E Fig).

The highly efficient single-cell genome amplification by multiple annealing- and looping-based amplification cycles (MALBAC) enabled us to detect as many 5fC sites as possible [27]. We focused on the 5fC on CpG sites named as 5fCpG. The 5fCpG calling scheme was basically according to our previous study to keep the 5fCpG sites calling consistent [22]. The stringent 5fC candidate pool was established by selecting CpG sites with ≥65% C-to-T ratio in at least two single-cell samples with malononitrile treatment, which was expected to remove potential polymerase chain reaction (PCR) errors. In the meantime, a “background pool” was defined by selecting sites with ≥65% C-to-T ratio in at least two untreated (negative control) single-cell samples, which was used to subtract additional background noises. Known SNP from dbSNP database hg19 v135 were removed from the candidate sites. The binomial test were used to identify 5fCpG candidate sites from our sequencing data, and CpG sites with Holm–Bonferroni (HB) method-adjusted *p* < 0.01 were selected as 5fCpG sites. A total of 24,830 to 45,398 5fCpG sites were identified from merged samples for each developmental stage analyzed (S1F Fig and Fig 1B). A total of 8,033 to 393,602 CpG sites were covered across all cells in each developmental stage at ≥3× sequencing depth, and 0.17% to 0.89% of them were 5fCpG sites by developmental stage aggregrated analysis (S1G Fig). That is, 0.17–0.89% of the CpG sites covered in every indivual cell had formyl modification detected in at least one individual cell of the same developmental stages. The percentage of 5fCpG in the genome of individual sperm was higher than that in the genome of individual oocyte, which was 0.065% and 0.050%, respectively (Fig 1C). The first polar body had a higher 5fCpG level (0.062%) than the oocyte, indicating the different extent of active demethylation occurred during oocyte maturation. After fertilization, the 5fCpG content drastically increased in pronuclei. At 16 to 18 h after fertilization, the male and female pronuclei presented comparable 5fCpG levels, 0.106% and 0.109%. Immunostaining of human zygotes using 5fC antibody also proved the existence of 5fC (Fig 1D). From the zygote to the 2-cell stage, the 5fCpG level decreased to 0.053% (Fig 1C). During the cleavage stages, a higher 5fCpG percentage was observed at the 8-cell stage (0.066%). The 5fCpG level of pluripotent hESCs (0.041%) was lower than that of the pluripotent inner cell mass (ICM, 0.062%). These results indicated that human early embryos exhibited extensive 5fC dynamics due to active demethylation.

### The distribution characteristics of 5fCpG in human early embryos

**Fig 2:**
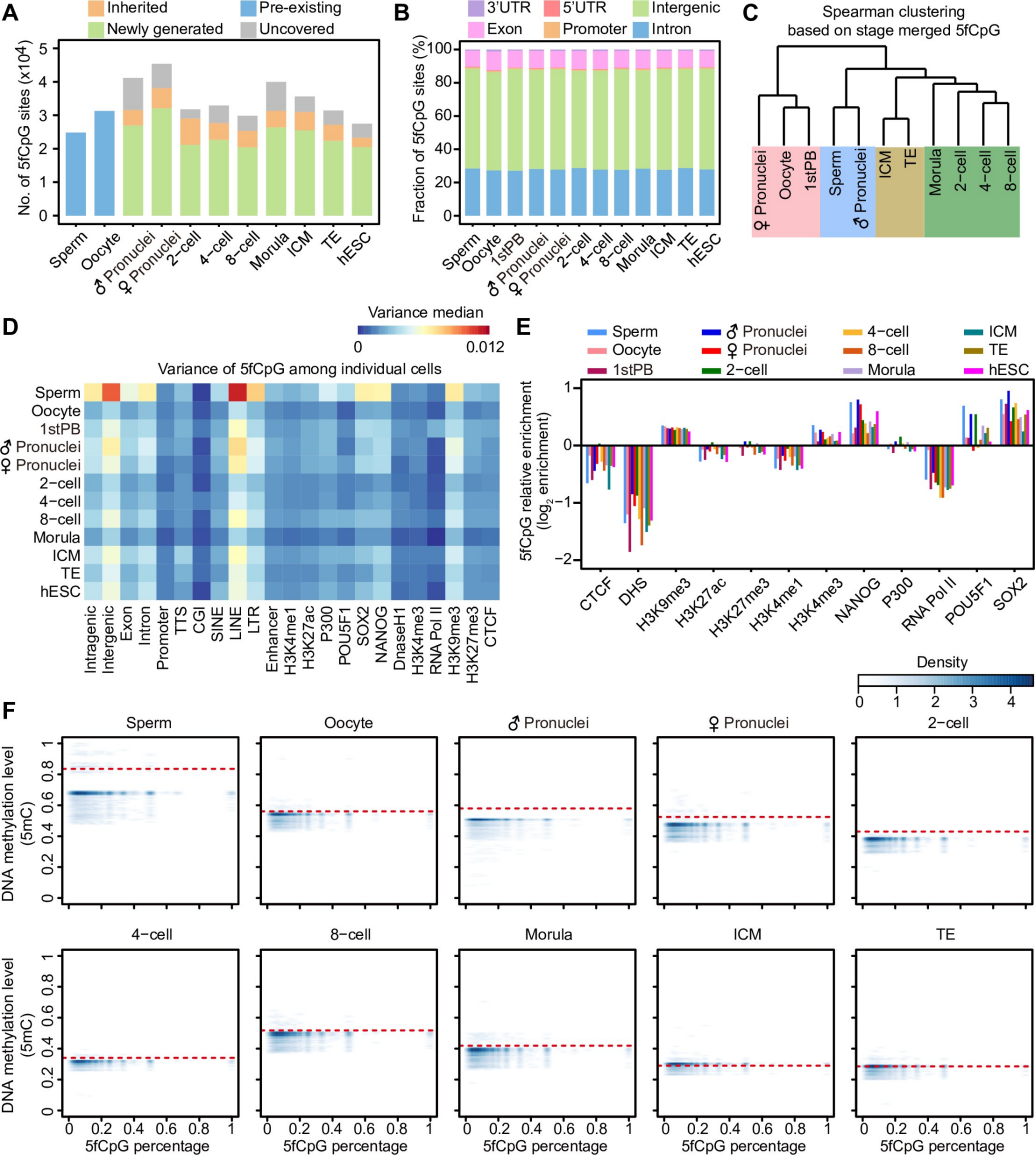
The production of 5fC in human early embryos. (A) The stacked bar plot showing the newly generated and inherited number of 5fCpG sites between two consecutive stages. The hESC was compared with ICM, whereas both ICM and TE were compared with morula. (B) The stacked bar plot showing the fraction of 5fCpG sites located in different genomic regions in each developmental stages. (C) Unsupervised clustering of stage merged 5fCpG sites calculated by Spearman correlation. (D) Heat map showing the median variance of 5fCpG abundance in different genomic regions among individual cells. The variance is calculated by the 5fCpG distribution in 1-kb window. The color key from blue to red represents the value of median variance from low to high. The binding peak of histone, transcription factor, and DNase I hyper-sensitive sites are downloaded from GSE29611, GSE61475, and GSE32970, respectively. (E) Relative enrichment analysis of 5fCpG sites in distinct binding regions of transcription factor and histone as well as DNase I hyper-sensitive sites. The DHS are downloaded from GSE32970. The binding peaks of histone and transcription factor are downloaded from GSE29611, GSE61475. In (A–E), the sample size in these panels are listed in Fig 1A. In (A–B) and (D–E), the numerical data is listed in S1 Data. (F) Density plot showing the relationship of 5mC and 5fC in 5fCpG-marked 1-kb windows. The x axis shows the 5fCpG percentage in 1-kb window calculated by the number of 5fCpG divided by the sum of unmodified CpG and 5fCpG covered. The red dashed line denotes the mean of DNA methylation level calculated in 1-kb windows across the genome in each developmental stage. The DNA methylaiton data of human early embryos are from GSE81233. The numerical data is listed in S2 Data. CGI, CpG island; CpG, cytosine phosphate guanine; CTCF, CCCTC-binding factor; DHS, DNase I hypersensitive sites; hESC, human embryonic stem cell; ICM, inner cell mass; LINE, long interspersed nuclear element; LTR, long terminal repeat; SINE, short interspersed nuclear element; TE, trophectoderm; TTS, transcriptional termination site; UTR, untranslated region; 5fC, 5-formylcytosine; 5fCpG, 5-formylcytosine phosphate guanine.

When we compared two consecutive developmental stages, most 5fCpG sites were newly generated, suggesting that the production of 5fCpG was highly dynamic during human early embryonic development (Fig 2A) [28]; 66%, 71%, and 67% of 5fCpG sites were newly generated in male pronuclei, female pronuclei, and 2-cell stages compared with those in sperm, oocyte and pronuclei stages, respectively. However, a certain proportion, 10% to 25% of 5fCpG, was inherited from the former developmental stage. Then we explored the relationship of newly generated 5fCpG in promoter regions and RNA expression of corresponsing genes (S2A Fig). Unlike in mice, we did not observe the phenomenon that 5fCpG production in promoters procedes the up-regulation of corresponding genes in the following human early embryo developmental stages [22]. Instead, the promoter 5fCpG-marked genes exhibited the trend of up-regulation in the current developmental stage, such as the oocytes, female pronuclei, 2-cell, 4-cell, 8-cell embryos, and TE when comparing to the former stage. Only 2,823 (10%) 5fCpG sites in hESCs were shared between ICM and hESCs, indicating drastically distinct active demethylation patterns between them (Fig 2A and S2B Fig). According to genomic annotation, over half of the 5fCpG sites were located in intergenic regions, and a steady portion of 5fCpG sites were situated in the intron (30% on average) and exon regions (10% on average) due to the relatively longer length of those elements (Fig 2B). However, relative enrichment analysis showed that 5fCpG sites were enriched in functional genomic regions, such as gene body regions, transcriptional termination sites (TTSs) and enhancers (S2C Fig), whereas these sites were depleted from intergenic regions, 5′ untranslated regions (UTRs), 3′UTRs, and CpG islands (CGIs). The enrichment of 5fCpG sites in promoters was variable at different developmental stages. In most stages except for the oocyte, 8-cell and trophectoderm (TE) stages, 5fCpG sites were depleted from the promoters. Then, we calculated the 5fCpG ratio in different genomic regions. In the intergenic region, the 5fCpG level was significantly higher than that in the intragenic region in the oocyte (*p* = 3.7 × 10^−2^), the first polar body (*p* = 2.0 × 10^−2^), four-cell (*p* = 1.3 × 10^−2^), ICM (*p* = 1.8 ×10^−2^), and hESC (*p* = 2.2 × 10^−3^) stages (S2D Fig). Furthermore, exon, intron, and TTS regions showed higher 5fCpG levels than did other genomic elements (S3A Fig). In contrast, CGI, except for the oocyte stage, showed relatively lower 5fCpG levels that coincided with their low DNA methylation (5mC) levels (S3A Fig). When promoters were classified into high-density CpG promoters (HCPs), intermediated-density CpG promoters (ICPs), and low-density CpG promoters (LCPs) according to CpG densities, the 5fCpG abundance in HCPs, ICPs, and LCPs was similar in most stages (S3B Fig). In 2-cell stage, the 5fCpG level in HCPs was lower than that in ICPs and LPCs (Student *t* test *p* = 5.0 × 10^−6^ and *p* = 6.9 × 10^−5^, respectively). Also in male pronuclei and 4-cell stage, the 5fCpG levels in HCPs were lower than that in LCPs (Student *t* test *p* = 3.0 × 10^−2^ and *p* = 4.5 × 10^−3^, respectively).

Then, we conducted unsupervised clustering of the embryos by 5fCpG sites (Fig 2C). The oocytes, the first polar bodies, and female pronuclei clustered together but were separated from other embryo stages. Sperm and male pronuclei also clustered together. Similarly, ICM and TE clustered together. The cleavage stages, from the 2-cell to morula stage, were clustered together. These results indicated the similarity and association of 5fCpG sites in neighboring developmental stages. To estimate the heterogeneity of the 5fCpG site distribution, we calculated the variance in the 5fCpG level in a consecutive 1-kb window in the genome among cells at each developmental stage [29]. The variance in sperm was highest, up to a median of 8.9 × 10^−3^ (S3C Fig). The variance in the morula stage was lowest, with a median of only 3.3 × 10^−3^. Then t-distributed stochastic neighbor embedding (t-SNE) analysis of 5fCpG-marked windows was performed at the window size of 1 kb, 10 kb, 100 kb, 1 Mb, 10 Mb, respectively. The cells at sperm and male pronuclei stages clustered relatively closely at the windows of 1 kb, 10 kb, and 1 Mb, while cells of different developmental stages dispersedly distributed in t-SNE map (S3D Fig). Similarly, principal component analyses (PCAs) were also conducted based on 5fCpG-marked windows. For the 1-kb and 10-kb windows, the heterogeneity among single cells of male pronuclei and female pronuclei was stronger than that of other stages (S4 Fig). For the window of 100 kb, 1 Mb, 10 Mb, the heterogeneity among single cells of male pronuclei and sperm was more dominant than that of other stages. The 5fCpG sites in the intergenic region exhibited higher variance compared with those in the intragenic region (Fig 2D). The variance in 5fCpG sites located in repeat element long interspersed nuclear elements (LINEs) was higher than that in short interspersed nuclear elements (SINEs) and long terminal repeats (LTRs). The binding sites obtaind from chromatin immunoprecipitation sequencing (ChIP-seq) data of hESC [30,31] was used as presumed ones in human early embryos. Heterochromatin regions (H3K9me3 marked) showed higher variance in 5fCpG sites than did regions marked by H3K4me3 and H3K27me3. The 5fCpG sites in promoters, CGIs, and RNA polymerase II binding sites showed relatively lower variance, suggesting a conserved 5fC distribution in functionally essential genomic regions. According to the published ChIP-seq data of hESCs [30,31], 5fCpG sites were enriched in heterochromatin regions marked by H3K9me3 and euchromatin regions marked by H3K4me3 (Fig 2E). However, these sites were depleted from the binding sites of CCCTC-binding factor (CTCF), and DNase I hypersensitive sites (DHS) in most preimplantation developmental stages. Additionally, 5fCpG were enriched in the binding sites of pluripotent transcription factors, such as NANOG, POU5F1, and SOX2. To explore the relationship between 5fCpG and 5mC, we calculated the DNA methylation level in 5fCpG-marked 1-kb windows in each developmental stage of human early embryos. Majority of 5fCpG-marked regions had relatively low methylation levels compared to the average 5mC level in the current stage in sperm, male pronuclei, female pronuclei, and 2 -cell (Fig 2F). However, this low DNA methylation tendency of 5fCpG-marked bins began to decrease since 4-cell onwards.

### 5fCpG distribution on repeat elements

**Fig 3:**
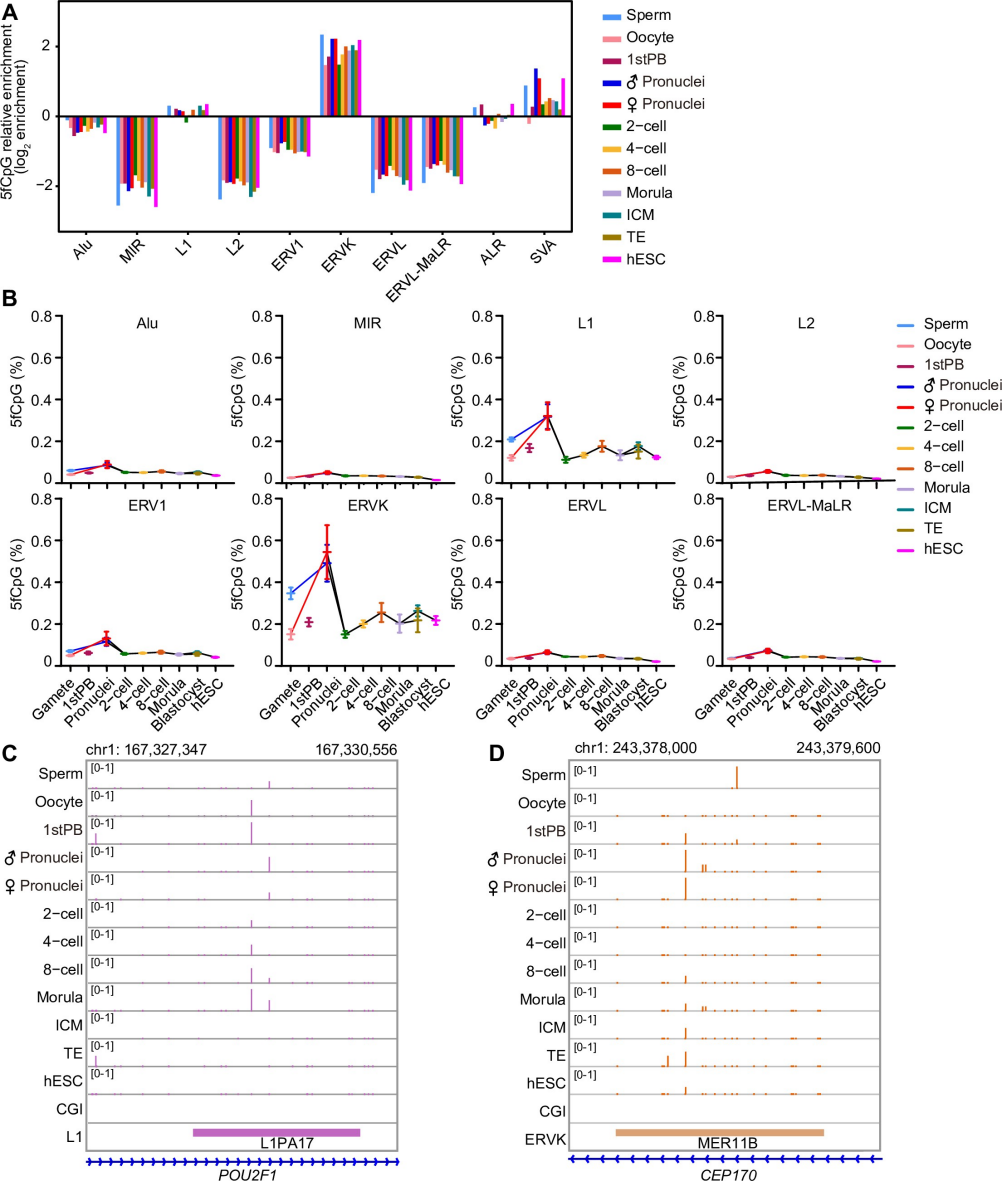
The 5fCpG distribution character on repeat elements. (A) Relative enrichment analysis of 5fCpG sites located in repeat elements and the subfamilies. (B) The line chart showing the 5fCpG level on subfamilies of repeat elements throughout the human early embryo developmental stages. The center is the mean of 5fCpG level and error bars are SEM. (A–B) The sample size in these panels are listed in Fig 1A, and the numerical data are listed in S1 Data. (C–D) The UCSC browser view of 5fCpG sites located in L1 (C) and ERVK (D). CGI, CpG island; ERVK, endogenous retrovirus-K; ERVL-MaLR, endogenous retrovirus-L, mammalian-apparent long-terminal repeat retrotransposon; hESC, human embryonic stem cell; ICM, inner cell mass; MIR, mammalian-wide interspersed repeat; PB, polar body; SEM, standard error of the mean; TE, trophectoderm; UCSC, University of California, Santa Cruz; 5fCpG, 5-formylcytosine phosphate guanine.

Repeat elements have been showed to participate in the regulation of early embryonic development [32]. Enrichment analysis showed that 5fCpG sites were enriched in LINEs and satellites compared with those in SINEs and LTRs (S5A Fig). Moreover, the 5fCpG levels in LINEs and the SINE/variable number of tandem repeats/Alu (SVA) were higher than that in other repeat elements (S5B Fig). For the subfamily of repeat elements, 5fCpG sites were relatively enriched in endogenous retrovirus-K (ERVK), L1 (except for two-cell stages) and SVA (except for oocyte stages; Fig 3A). The level of 5fCpG was higher in L1, the evolutionarily younger subfamily of LINEs, compared with that in L2, the evolutionarily older subfamily (Fig 3B and 3C). The subfamilies of SINEs, Alu, and mammalian-wide interspersed repeat (MIR), showed similar 5fCpG levels. The 5fCpG level in ERVK was also higher than that in other subfamilies of LTRs (Fig 3B and 3D). Then we explored the RNA expression and DNA methylation level on L1 and ERVK with 5fCpG modification. Compared with the randomly selected ones only with unmodified CpG, these 5fCpG-marked L1 showed lower expression level in most developmental stages (S5C Fig). In ERVK, the 5fCpG-marked ones exhibited lower expression level in 2-cell embryos, morula, and TE but higher expression level in oocytes and 4-cell embryos. The markedly high 5fCpG level in L1 and ERVK may be associated with the regulation of the transcription of these repeat elements. The 5fCpG-marked L1 and ERVK showed significantly higher DNA methylation levels compared to the randomly selected ones (S5D Fig), which suggested that those repeat element regions with higher DNA methylation modification had higher tendency to retain the active demethylation marks.

### Comparison of 5fCpG in paternal and maternal genomes

**Fig 4:**
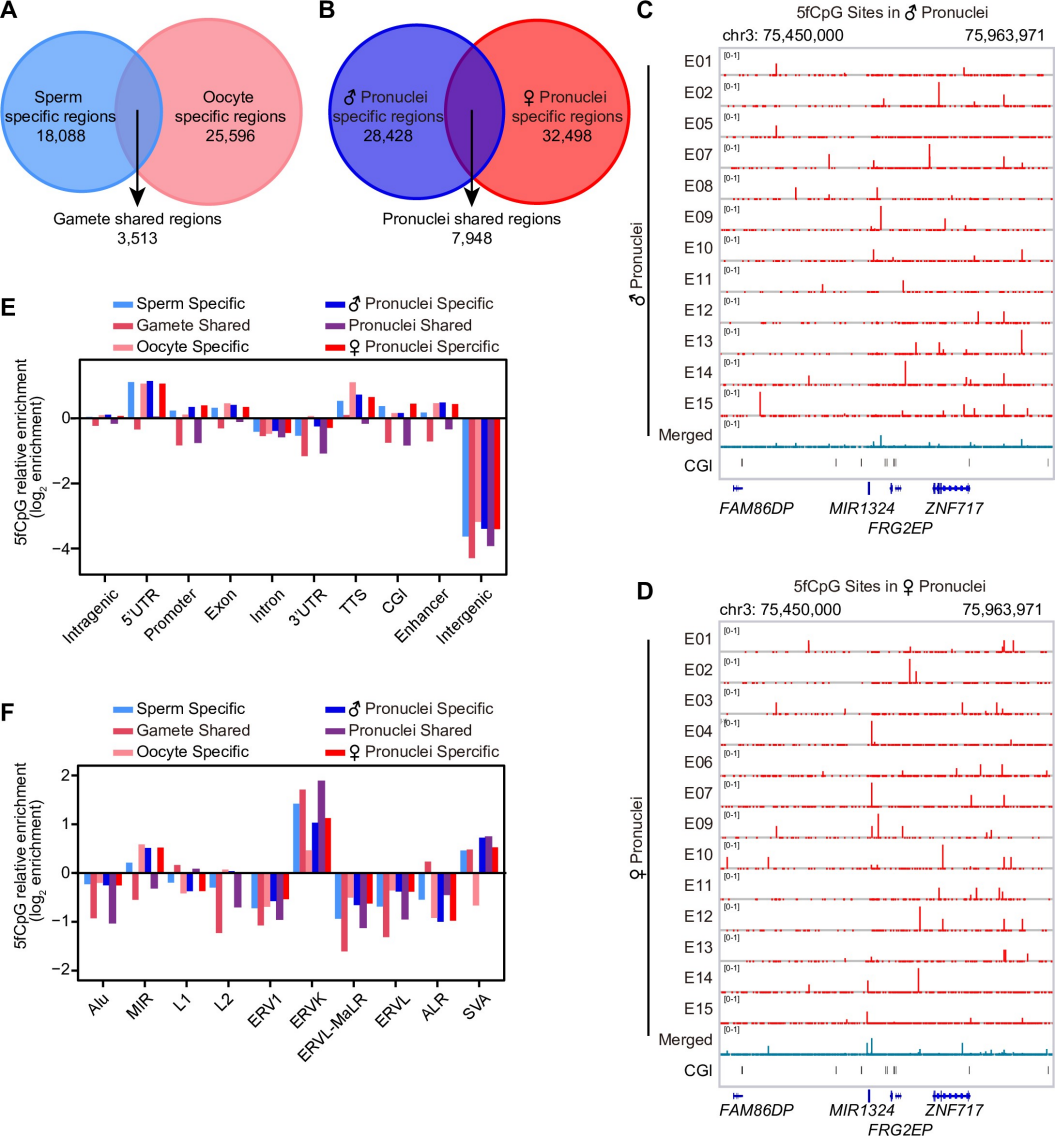
The features of 5fCpG sites distribution on paternal and maternal genome. (A) Venn diagram showing the overlap of 5fCpG-marked regions (1-kb window) between sperm and oocyte. The number of sperm-specific, oocyte-specific, and gamete-shared 5fCpG-marked regions are listed in the diagram. (B) Venn diagram showing the overlap of 5fCpG-marked regions (1-kb window) between male pronuclei and female pronuclei. The number of male pronuclei-specific, female pronuclei-specific, and pronuclei-shared 5fCpG-marked regions are listed in the diagram. (A–B) The sample size in these panels are listed in Fig 1A. (C–D) The UCSC browser view of 5fCpG sites in individual male pronucleus (C) and female pronucleus (D). (E) Relative enrichment of gamete-specific, gamete-shared, pronuclei-specific, and pronuclei-shared 5fCpG-marked regions (1-kb window) in distinct genomic region. (F) Relative enrichment of gamete-specific, gamete-shared, pronuclei-specific, and pronuclei-shared 5fCpG-marked regions (1-kb window) in repeat elements and subfamilies. (E–F) The sample size in these panels are listed in Fig 1A, and the numerical data are listed in S1 Data. CGI, CpG island; MIR, mammalian-wide interspersed repeat; TTS, transcriptional termination site; UCSC, University of California, Santa Cruz; UTR, untranslated region; 5fCpG, 5-formylcytosine phosphate guanine.

The parental genomes undergo different extents of demethylation and remethylation during human embryonic development [12,33]. To determine the 5fCpG features of parental genomes, we compared the 5fCpG-marked regions defined in which 1-kb consecutive windows in genome had at least one 5fCpG sites in gametes and pronuclei. A total of 3,513 (10%) 5fCpG-marked regions in oocytes were shared with those in sperm (Fig 4A). Additionally, 7,948 (20%) 5fCpG sites in female pronuclei were shared with those in male pronuclei (Fig 4B, 4C and 4D). Enrichment analysis showed that in contrast to gamete- and pronuclei-shared regions, gamete-specific and pronuclei-specific 5fCpG-marked regions were enriched in promoters, exons, CGI, and enhancers (Fig 4E). The gamete-shared and pronuclei-shared 5fCpG-marked regions were enriched in LINEs and satellites (S5E Fig), especially in L1, and depleted in MIR compared with gamete-specific and pronuclei-specific regions (Fig 4F). These results suggested that 5fCpG was distinctly distributed on parental genomes.

### Variable differences of 5fCpG levels in paired pronuclei

**Fig 5:**
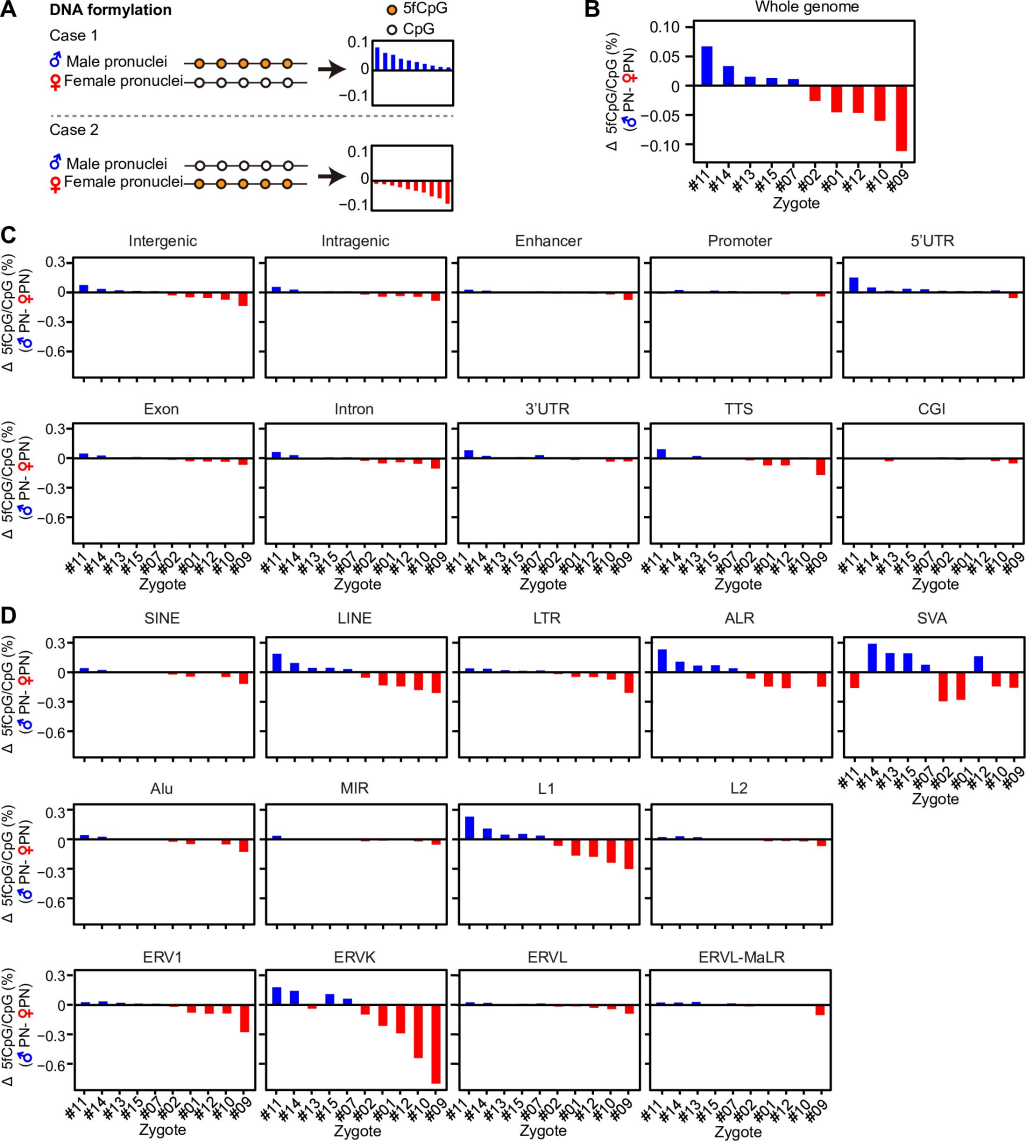
The asymmetric 5fCpG distribution in paired pronucleus. (A) The illustration diagram showing the two cases of 5fCpG level in paired pronucleus. The bar plot is used to show the difference of 5fCpG level in male pronuclei minus that in corresponding female one. The bar is in blue if the male pronuclei has higher 5fCpG level than female one (difference > 0), otherwise it is in red. (B) The bar plot showing the difference of 5fCpG level between paired pronucleus in whole genome (*n* = 10). The difference is calculated by 5fCpG level in male pronuclei minus that in corresponding female one. The order of paired pronuclei is ranked by the value of difference from high to low. (C) The bar plot showing the difference of 5fCpG level between paired pronucleus in distinct genomic region (*n* = 10). The pronuclei are ranked by the order showed in Fig 5B. (D) The bar plot showing the difference of 5fCpG level between paired pronuclei in distinct repeat elements (*n* = 10). The pronuclei are ranked by the order showed in Fig 5B. (B–D) The numerical data are listed in S1 Data. ALR, alpha-satellite repeat; CGI, CpG island; CpG, cytosine phosphate guanine; ERV1, endogenous retrovirus-1; ERVK, endogenous retrovirus-K; ERVL, endogenous retrovirus-L; ERVL-MaLR, endogenous retrovirus-L, mammalian-apparent long-terminal repeat retrotransposon; LINE, long interspersed nuclear element; LTR, long terminal repeat; MIR, mammalian-wide interspersed repeat; SINE, short interspersed nuclear element; SVA, SINE/variable number of tandem repeats/Alu; TTS, transcriptional termination site; UTR, untranslated region; 5fCpG, 5-formylcytosine phosphate guanine.

In the pronuclei stage, the 5fCpG level is higher than that in other stages, which is similar in mice [22]. In mice, for paired pronuclei, the 5fCpG levels of male pronuclei are always higher than those of paired female pronuclei. However, in humans, paired pronuclei showed a distinct pattern. In total, we successfully obtained 10 paired pronuclei to quantify 5fCpG levels. Five pairs presented higher 5fCpG levels in male pronuclei, and the highest difference was up to 0.067% (Fig 5A and 5B). In contrast, in the remaining 5 pairs, female pronuclei had higher 5fCpG levels than did paired male pronuclei. That is, in some zygotes the 5fCpG levels of paternal gemone were higher than that of maternal genome, whereas in others it was opposite. To a certain extent, the differences in both intergenic and intragenic regions collectively contributed to the differences in the whole genome (Fig 5C). However, the difference in 5fCpG levels in enhancers and promoters between paired parental nuclei was nonsignificant. The most drastic differences in paired pronuclei resulted from the repeat elements, LINEs and ALRs (Fig 5D). In particular, the subfamilies L1 and ERVK showed the most significant differences. The variable differences of 5fCpG levels in paired pronuclei were completely different from those in mice, mainly resulting from the difference in 5fCpG on repeat elements.

### 5fCpG levels in paired two-cell embryos

For paired blastomeres in two-cell embryos, we compared 5fCpG levels between two blastomeres from the same embryo. Three pairs of two-cell blastomeres showed different 5fCpG levels, and one pair showed similar levels (S6A Fig). Additionally, the difference in 5fCpG levels was maintained in enhancers, promoters, exons, and introns (S6B Fig). For subfamilies of repeat elements, these four pairs of blastomeres showed similar 5fCpG levels on Alu, MIR, L2, ERV1, ERVL, and ERVL-MaLR (S6C Fig). However, the difference in 5fCpG distribution was mainly exhibited in L1 and ERVK, subfamilies of LINEs, and LTRs, respectively, which resembled the situation in paired pronuclei (Fig 5D).

### Regulatory elements enriched in 5fC-marked regions

**Fig 6:**
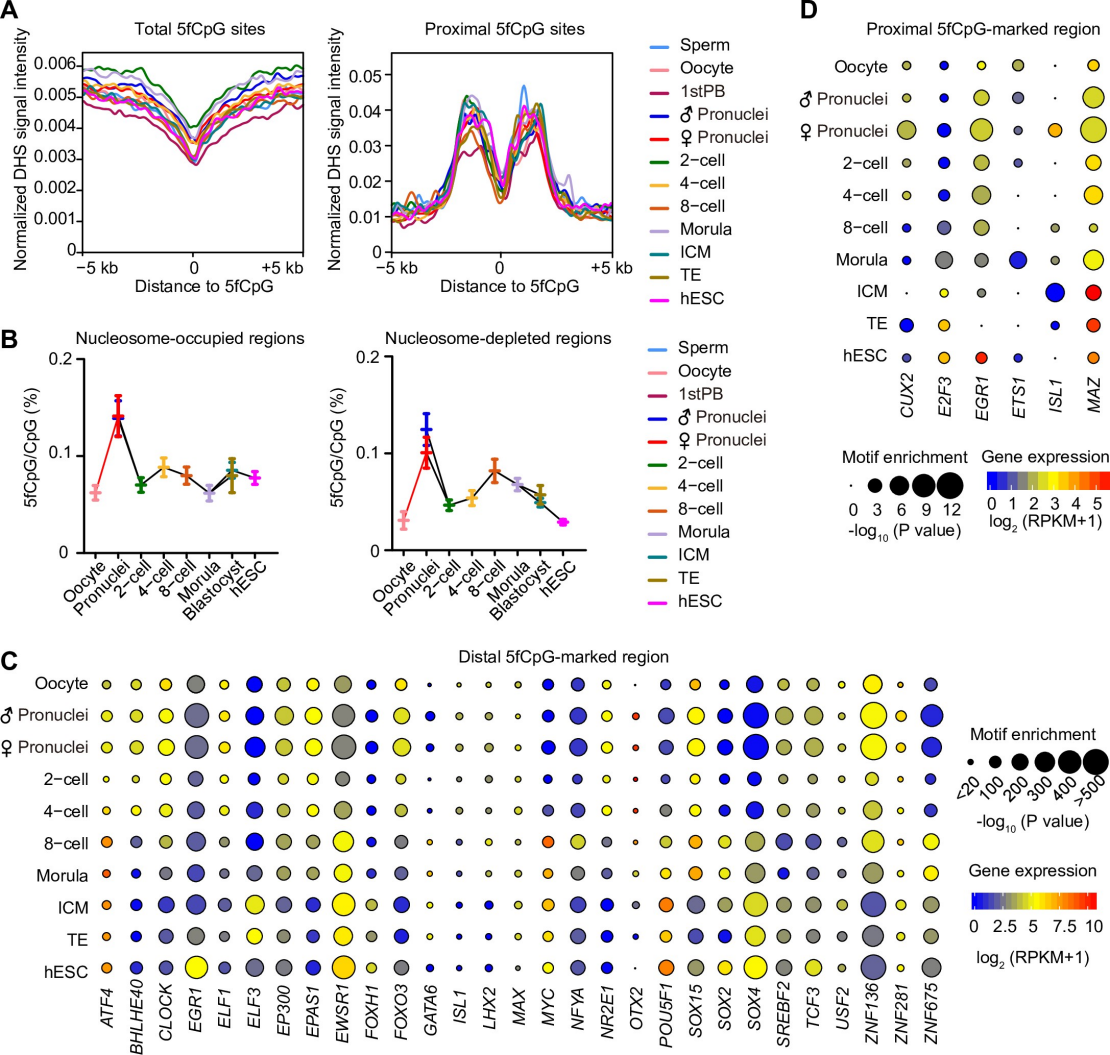
The regulatory network of 5fC-mark region. (A) The normalized DHS signal of hESC at the center of all 5fCpG sites and its flanking regions (left panel) as well as at the center of proximal 5fCpG sites (TSS ± 2 kb) and its flanking regions (right panel) in each development stage are shown. The DHS signal of hESC is downloaded from GSE32970. (B) The line chart showing the 5fCpG level in NORs (left panel) and in NDRs (right panel). The 5fCpG level was calculated by the number of 5fCpG sites divided by the sum number of 5fCpG and unmodified CpG sites. scCOOL-seq of human preimplantation embryos is from GSE100272. The center is the mean of 5fCpG level, and error bars are SEM. (C) Motif analysis of 5fCpG-marked distal regions (2 kb away from TSS). Only motif with *P* ≤ 10^−20^ and RPKM ≥ 1 in at least one stage are shown in the diagram. The significance was calculated by the binomial test in HOMER by default of motif enrichment. The color of the circle from blue to red indicates the expression level in each stage from low to high. The scRNA-seq data of human early embryos and ESCs are from GSE36552. The size of the circle indicates the −log_10_(*P* value). (D) Motif analysis of 5fCpG-marked proximal regions (TSS ± 2 kb). Only motif with *P* ≤ 10^−5^ and RPKM ≥ 1 in at least one stage are shown in the diagram. The significance was calculated by the binomial test in HOMER by default of motif enrichment. The color of the circle from blue to red indicates the expression level in each stage from low to high. The scRNA-seq data of human early embryos, and ESCs are from GSE36552. The size of the circle indicates the −log_10_ *P* value. (A–D) The sample size in these panels are listed in Fig 1A. (B–D) The numerical data are listed in S1 Data. CpG, cytosine phosphate guanine; DHS, DNase I hypersensitive sites; ESC, embryonic stem cell; hESC, human embryonic stem cell; HOMER, hypergeometric optimization of motif enrichment; NDR, nucleosome-depleted region; NOR, nucleosome-occupied region; scCOOL-seq, chromatin overall omic-scale landscape sequencing; RPKM, reads per kilobase of transcript per million mapped reads; scRNA-seq, single-cell RNA sequencing; SEM, standard error of the mean; TSS, transcription start site; 5fC, 5-formylcytosine; 5fCpG, 5-formylcytosine phosphate guanine.

Taking advantage of the DNase-seq data of hESCs as putative DHS of human pluripotent ICM at the blastocyst stage [34,35], we analyzed the chromatin state surrounding 5fC sites. Throughout all developmental stages we analyzed, compared with its flanking regions, the center of 5fC sites showed relatively less chromatin accessibility (Fig 6A). For the proximal 5fC sites (within TSS ± 2 kb), this phenomenon was even more prominent. However, the shore regions (approximately 1392 base pairs [bp] upstream or downstream of TSS) around the center showed strong DHS signals (Fig 6A). Using the chromatin overall omic-scale landscape sequencing (scCOOL-seq) data of human early embryos [7], we analyzed the 5fCpG levels on the nucleosome-occupied regions and nucleosome-depleted regions (NDRs; Fig 6B). Interestingly, the 5fCpG level in the nucleosome-occupied regions was higher than that in NDRs in most developmental stages, such as the oocyte (*p* = 1.5 × 10^−2^), two-cell (*p* = 2.2 × 10^−2^), four-cell (*p* = 1.2 × 10^−2^), ICM (*p* = 1.3 × 10^−3^), and hESC (*p* = 5.9 × 10^−6^) stages. This finding is compatible with previously published results showing that 5fCpG could interact with histones and organize nucleosome positioning [18,36].

According to previous studies, 5fCpG on tissue-specific enhancers is associated with the up-regulation of the expression of corresponding genes [18,37]. We performed motif analysis of distal and proximal 5fCpG-marked genomic regions to find out the enriched transcription factors in these regulatory regions. In the distal 5fCpG-marked regions (2 kb away from TSS), we found that the enhancer-associated protein EP300 that was expressed across the human preimplantation devleopment, which acts as histone acetyltransferase to regulate transcription and is vital for embryonic development and cell proliferation [38,39], showed consistant enrichment in distal 5fCpG-marked regions (Fig 6C). The binding motif of EWSR1 involving in mitosis [40] and neuronal morphology [41] was strongly enriched in distal 5fCpG-marked regions with gradually increased expression across all stages analyzed. ZNF136, ZNF281, and ZNF675, the members of zinc-finger family, also exhibited significant enrichment in these regions. Besides, the binding motifs of pluripotency transcription factors, such as POU5F1 and SOX2, were significantly enriched. Moreover, the binding motifs of several transcription factors that determined cell fate during early lineage differentiation, such as SOX4, SOX15, and GATA6, were also enriched in these distal 5fC-marked regions. For the proximal 5fCpG-marked genomic regions (TSS ± 2 kb), the binding sequences of transcription factors regulating the cell cycle and mitosis, such as E2F3 and EGR1, were enriched (Fig 6D). Additionally, the binding motif of MAZ, a transcription factor regulating transcription complex formation which was expressed in all stages analyzed, was consistently enriched in the proximal 5fCpG-marked regions across human early embryonic development. Collectively, these analyses demonstrated that 5fCpG-marked regulatory regions exhibited the tendency of important transcription factors’ binding during human early embryonic development.

## Discussion

After fertilization, embryos undergo drastic epigenomic reprogramming, especially TET-mediated DNA demethylation. Here, we used single-cell CLEVER-seq to depict the DNA formylation profiles of human early embryos. During preimplantation development in humans, pronuclei (16–18 h after intracytoplasmic sperm injection [ICSI]) exhibited the highest 5fCpG levels. Male pronuclei and female pronuclei showed comparable 5fCpG levels, although the paternal genome experienced a much more extensive demethylation process from sperm to male pronuclei compared to maternal genome [12]. Furthermore, we analyzed 10 pairs of pronuclei from the same zygotes and found variable 5fCpG content in pronuclei. That is, in some zygotes, 5fCpG levels were higher in male pronuclei than in female ones, whereas in other zygotes it was the opposite. Different from mice, in 5 pairs of human pronuclei, the 5fCpG level in male pronuclei was higher than that in paired female pronuclei; the remaining 5 pairs showed the opposite phenomenon that female pronuclei had a higher 5fCpG level than did male pronuclei. Because *TDG* is extremely lowly expressed in human early embryos, it may not account for the inconsistent 5fCpG level in paired pronuclei [5,42]. Using immunostaining, our previous work showed that the 5hmC distribution in male and female pronuclei of human was also inconsistent: although majority of male pronuclei showed stronger 5hmC signals, still some female pronuclei exhibited higher fluorescent intensity of 5hmC compared with paired male one [5]. In mice zygotes, the 5fC and 5hmC level in paternal genome was always higher than that in maternal genome in the same embryo [22,43]. Different from mice, the active demethylation extent between parental genome in human zygotes may present in an embryo-specific manner due to the complicated genetic background of human embryos. The obvious difference in 5fCpG in paired pronuclei came from the 5fCpG in repeat element L1 and ERVK, the subfamilies of LINEs and LTRs. In addition to pronuclei, early embryos in other stages showed relatively higher 5fCpG abundance in L1 and ERVK. 5fC could reduce the specificity of the substrate to RNA polymerase II; thus, the high level of 5fC in those repeat elements may reduce their transcription to improve genome stability [44,45].

The formyl group of 5fC has high activity to react with the amine residues of protein to form the Schiff base complex [18]. The transcription regulators selectively binding to 5fC had been identified by proteomics screening, such as FOXK1, FOXP1, and FOXI3 [19]. In human embryos, we also found that the binding motifs of essential transcription factors, such as POU5F1 and SOX2 to maintain pluripotency and E2F3 and EGR1 to regulate the cell cycle, were significantly enriched in 5fCpG-marked regulatory regions. In addition to transcription factors, the lysines of histone proteins can interact with the formyl group of 5fC [36,46]. Consistent with the above data, our data showed that the 5fCpG level in nucleosome-occupied regions was higher than that in nucleosome-depleted regions in many developmental stages. The function of 5fC in organizing nucleosome position and altering the structure of the DNA helix could contribute to transcription control [18,36,47]. Increasing evidence shows that 5fC does not only serve as an intermediate of active DNA demethylation but also participates in functional regulations [48]. In the future, functional work is needed to determine the comprehensive roles of 5fC in vivo.

For a better understanding of the complicated active DNA demethylation process after fertilization, the features of 5hmC and 5caC also remain to be investigated, which rely on the improvement of techniques to accurately quantify these DNA modifications [49–52]. In summary, our work presents the 5fC landscapes of human early embryos, which provides new insights into the complex regulation network during human preimplantation development.

## Methods

### Ethics statement

The research project was approved by the Reproductive Study Ethics Committee of Peking University Third Hospital (research license number 2017SZ-081), and the study strictly followed the guidelines set forth by the Ethics Committee. Oocytes were voluntarily donated by female donors at the Center for Reproductive Medicine, Peking University Third Hospital, with signed written informed consent. Sperm donation was provided by a healthy man with proven fertility who signed the written informed consent.

Standard in vitro fertilisation (IVF) protocols for the gametes and embryo collection, thawing, and culture were executed as previously described [53,54].

### Collection of human gamete and early embryos

#### Gametes and the first polar body

After several rounds of washing with HTF medium (Life global), the swim-up sperm were collected for further processing. Motile single sperm with normal morphology was collected via micromanipulation. For the metaphase II oocyte, the granulosa cells were removed with the treatment of hyaluronidase (Sigma). The first polar bodies were collected by a micropipette with laser-assisted biopsy. The metaphase II oocytes without the first polar body were transferred into an acidic solution drop (1 μL 36% HCl diluted in 1 mL DPBS) to remove the zona pellucida. The oocytes were then washed several times in DPBS with 0.1% HSA by gentle pipetting to eliminate any somatic contamination.

#### Human preimplantation embryos

The assessment of embryos development and the scheduling of embryo collection were performed according to a previous study [55]. Following oocyte retrieval, oocytes were fertilized via ICSI and signs of fertilization were checked on the following day. Male and female pronuclei of zygotes were collected through careful micromanipulation after broken the zona pellucida and the cytoplasmic membrane. The 2-cell and 4-cell embryos were collected at 27 h and 48 h post fertilization, respectively. The 8-cell and morula stage embryos were obtained at Day 3 and Day 4 according to their morphology, respectively. At Days 5 and 6, the ICM and TE of blastocysts were isolated with laser-assisted micromanipulation. Embryos with developmental retardation were excluded from this study. The embryos were treated with acidic solution (1 μL 36% HCl diluted in 1 mL DPBS) to remove the zona pellucida. The embryos without zona pellucida were then washed several times by gentle pipetting in DPBS with 0.1% HSA. Subsequently, a mixture of Accutase (Sigma) and 0.25% trypsin at the ratio of 1:1 was used to digest the embryo into single cells in humidified incubator for 20 to 50 min with 5% CO_2_ at 37°C. Then single blastomeres were carefully washed in DPBS with 0.1% HSA before being transferred into lysis buffer.

### hESC culture

The hESCs (H9) were purchased from WiCell Institute. hESCs were cultured on the hESC-Qualified Matrix (Corning) in a feeder-free condition. And the complete E8 culture medium were used in cell culture. With regular passaging, we collected the colonies of hESCs and digested them into single-cell suspension using Accutase (Sigma) at 37°C for 1 h. Then the single cells of hESCs were used for CLEVER-seq library construction.

### CLEVER-seq library construction

The CLEVER-seq experiment was carried out according to the previous study with slight modification [22]. The single cells of human early embryo were transferred into lysis buffer (20 mM Tris, 2 mM EDTA, 20 mM KCl, and 0.3% TritonX-100, 1 mg/mL QIAGEN protease) by mouth pipette. After centrifugation (1 min at 7,000 r.p.m., 4°C), the sample was incubated at 50°C for 3 h to release the genomic DNA followed by 70°C for 30 min to inactive the protease. Then trace mount of spike-in DNA contain 5fC modification was added into sample. In order to label the 5fC, 150 mM malononitrile (J&K) was used. Mineral oil (15 μL) was added onto the surface of the sample to prevent evaporation. The sample was incubated at 37°C for 20 h with constant shaking at 850 r.p.m. in Thermomixer (Eppendorf). Then the single cell genome was amplified by MALBAC (Yikon Genomics) according to the user guide. After amplification, the genomic DNA was fragment into approximately 300 by covaris S2. The library construction was performed using KAPA Hyper Prep Kit (Kapa Biosystems). The libraries were sequenced on Illumina Hiseq 4000 platform in pair-end 150 bp model (Novogene).

### Genomic DNA sequencing

In order to differentiate the male and female pronucleus, the genomic DNA was sequenced from the peripheral blood and sperm pellet of couple donators to call SNP. The genomic DNA was extracted using DNesay Blood and Tissue Kit (Qiagen). About 500 ng DNA was fragment into 300 bp by by covaris S2. The libraries were constructed using KAPA Hyper Prep Kit (Kapa Biosystems). And the libraries were sequenced the same way as CLEVER-seq libraries.

### Immunostaining of 5fC and 5mC in human zygote

The immunostaining was carried out according to previous study with slight modification [5]. Briefly, the zona pellucida of human zygotes was removed by treating with acidic solution (1 μL 36% HCl diluted in 1 mL DPBS). After several times of washing in DPBS, the sample was fixed 4% paraformaldehyde for 30 min at room temperature. After washing with DPBS containing 0.1% HSA, the membrane was permeated in 1% Triton X-100 at room temperature for 15 min. For detection of 5fC and 5mC, the DNA of embryos was denatured by 4N HCl at room temperature for 20 min and then neutralized for 10 min with 100 mM Tris-HCl. The sample was blocked in 1% goat serum at room temperature for 1.5 h. The primary antibody of 5fC (Active Motif, Cat#61223) and 5mC (Eurogentec, Cat#BI-MECY-1000) was diluted at the ratio of 1:500. The sample was incubated in the diluted antibody buffer for 3 h at room temperature. After washing, the sample was incubated with Alexa Fluor 594 goat anti-rabbit IgG (1:500, Invitrogen, Cat#A-11012) and Alexa Fluor 488 goat anti-mouse IgG (1:500, Invitrogen, Cat#A-11001) for 1 h at room temperature. All the images were captured and analysed using Nikon A1R high-speed laser confocal microscopy.

### Reads quality control and alignment

We used Trim Galore (version 0.3.3) to remove low quality bases, adaptor, and MALBAC primer sequences. Then, reads passed quality control were mapped to human reference genome (version: hg19) using Bismark (version 0.13.0) [56]. Then PCR duplicates were removed with the command ‘samtools rmdup’ (version 0.1.18) [57]. A 137-bp model DNA contain 5fC modification (model 1 or model 2; information are provided in S2 Table), and lambda were added in same library to estimate the C-to-T conversion rate and random C-to-T conversion rate in each single cell experiment.

### CNV detection with CLEVER-seq data

First, we selected euploid cells for downstream analysis. Briefly, read counts of 1 Mb nonoverlapping windows were used to deduce CNV by R package HMMcopy [58] with GC and mappability correction.

### Gender estimation of pronuclei

With read counts of sex chromosomes, we can estimate gender of each embryo. In this way, we can unambiguously distinguish between male PN and female PN in paired pronuclei having chromosomes XY, also male PN from unpaired pronuclei having chromosomes XY. As for pronuclei having chromosomes XX, we used SNPs identified from parental genomic DNA to distinguish between male PN and female PN. Using an established pipline [12,59], parental genomic data were cleaned with Trim Galore (version 0.3.3) and mapped to the human reference genome hg19 with bwa (version 0.7.12) [60]. PCR duplications were removed with Picard (version 1.126); then SNPs were identified with GATK HaplotypeCaller [61].

### 5fCpG site identification and 5fCpG level estimation

Following a previous study [22], we restricted 5fC at CpG sites without known SNPs (dbSNP database hg19 version 135). All CpG sites with C-to-T conversion rate ≥0.65 in at least two treated cells were grouped as the candidate pool to avoid PCR amplification errors, whereas CpG sites with C-to-T conversion rate ≥0.65 in control cells were grouped as the candidate noise pool to avoid background noises. For each treated single cell, CpG sites covered ≥3 times with C-to-T conversion rate ≥0.65, in the candidate pool but not in the noise pool, were identified as candidate 5fCpG sites in this single cell. To calculate the possibility of observing 5fCpG by chance, we used binomial distribution estimation with ‘dbinom’ in R, with C-to-T conversion rate estimated from lambda DNA, according to previous study [62]. For each site, the number of reads supporting ‘T’ were denoted as ‘NT’ and the number of reads supporting ‘C’ were denoted as ‘NC’, and the sequencing depth was NT+NC. 5fCpG sites with Holm–Bonferroni method-adjusted *p* < 0.01 were retained as final 5fCpG sites in this single cell for further analysis. And CpG sites covered ≥3 times with C-to-T conversion rate ≤0.25 were identified as unmodified C sites. In order to model false positives, the negative control single cells (those not treated with malononitrile) were also analyzed with the same procedures. The number of called 5fCpG sites were divided by the number of total sequenced CpG sites (defined as ‘5fCpG abundance’) for both the treated and untreated samples. And false-positive detection rate was calculated by dividing the 5fCpG abundance of the untreated samples by that of the treated samples. At the C-to-T cut-off of 0.65, false-positive detection rate of 8% was observed in hESC samples.

For random C-to-T rate of lambda DNA, we selected the C sites covered ≥4 times in an individual cell sample to avoid sequencing errors and counted the number of T readout on these C sites. The random C-to-T rate was calculated by the number of T readouts divided by the total number of these C sites sequenced. Based on labmda DNA, the average false positive rate in 130 treatment sample was 1.15%.

The cluster and the variance of 5fCpG level were calculated based on 1-kb windows across the genome, and only windows with 5fCpG sites identified in at least one stage were retained for this analysis. The motif enrichment analysis was done with HOMER (version 4.10.3) based on regions 100 bp upstream and 100 bp downstream of 5fCpG sites identified in each stage.

The global or genomic region 5fCpG ratio was estimated by the number of 5fCpG sites divided by the sum of the number of 5fCpG and unmodified C sites. The genomic locations of exons, introns, CGIs, 5′UTR, and 3′UTR repeat elements and their subfamilies were downloaded from the UCSC genome browser. Promoters were regions 1-kb upstream and 0.5-kb downstream of TSS, which were grouped into three classes based on CpG dengsity, that is, HCP, ICP, and LCP [63]. The significance of 5fCpG level between genomic regions was calculated by two-tailed Student *t* test.

The published data sets used in this study were downloaded from the Gene Expression Omnibus (GEO) with the following accession numbers: GSE100272 (scCOOL-seq of human preimplantation embryos), GSE36552 (scRNA-seq data of human preimplantation embryos), GSE32970 (DNase-seq peak and signal of human ESCs), GSE29611 (ChIP-seq of human ESCs), GSE61475 (transcription factor binding sites in human ESCs).

### DNA methylation levels and RNA expression levels of 5fCpG marked repeats

We used read counts located in each 5fC marked repeat region and RPKM (reads per kilobase of transcript per million mapped reads) method to estimate the expression levels of repeat elements. As a control, we randomly selected the same number of L1 or ERVK regions with only unmodified CpG covered to calculate the corresponding DNA methylation levels or RNA expression levels. The repeat annotation is downloaded from hg19 Repeat Masker in UCSC. The RNA-seq data are from GSE36552, and the DNA methylation data are from GSE81233.

### t-SNE and PCA analysis of 5fCpG sites

5fCpG percentage in 1-kb, 10-kb, 100-kb, 1-Mb, or 10-Mb windows were calculated by the number of 5fCpG divided by the sum of unmodified CpG and 5fCpG. Only the windows containing 5fCpG modification at least in one single cell were considered when performing dimensionality reduction analysis. PCA was performed by ‘pcaMethods’ package in R, and tSNE analysis was performed by ‘tsne’ package in R.

### Code availability

All the data analysis was conducted in perl, Python, and R language. All the computational code used in this study are available upon contacting with corresponding authors.

## Supporting information

S1 Fig Quality control of CLEVER-seq library and the summary of the number of 5fCpG sites. (A) Representative copy number variation plot showing that only euploid cell were retained for further analysis in this study. (B) Box plot showing the C-to-T conversion rate of spike-in oligo DNA which contained a 5fC modification in each stages. The box indicates the median, 25% quartile, and 75% quartile. The whisker represents the 1.5 times of IQR. (C) Box plot showing the mapping rate of 130 libraries in each stages. The box indicates the median, 25% quartile, and 75% quartile. The whisker represents the 1.5 times of IQR. (D) Box plot showing the CpG coverage at 1× (blue), 3× (red), 5× (green) depth of libraries in different stages. The box indicates the median, 25% quartile, and 75% quartile. The whisker represents the 1.5 times of IQR. (E) Histogram showing the number of covered unmodified CpG sites in each stages at 3× depth. (B–E) The sample size in these panels are listed in Fig 1A; the numerical data are listed in S1 Table. (F) The table showing the number of 5fCpG sites identified in each single cell and the number of stage-merged 5fCpG sites. (G) The table summarizing the number of CpG sites covered by all cells in each stage at 3× depth, the number as well as the ratio of 5fCpG sites in all covered CpG sites. CLEVER-seq, chemical-labeling-enabled C-to-T conversion sequencing; CpG, chemical-labeling-enabled C-to-T conversion sequencing; IQR, interquartile range; 5fC, 5-formylcytosine; 5fCpG, 5-formylcytosine phosphate guanine.
(TIF)

S2 Fig The 5fCpG enrichment on genomic region and comparison between ICM and hESC. (A) The normalized fold changes of average gene expression levels between two consecutive stages for genes with 5fCpG in promoters (TSS ± 2 kb). The hESC was compared with ICM, whereas both ICM and TE were compared with morula.The color from blue to red indicate the values of log_2_ of expression level fold change between two consecutive stages from low to high. The scRNA-seq data of human early embryos are from GSE36552. (B) Venn diagram showing the 5fCpG site overlap between ICM and hESC. The number of ICM-specific, hESC-specific, and their shared 5fCpG sites are listed in the diagram. (C) Bar plot showing the relative enrichment of 5fCpG sites on different genomic region. (D) Line chart showing the 5fCpG level in intergenic and intragenic region across human early embryo developmental stages. The center is the mean of 5fCpG level and error bars are SEM. (A–D) The sample size in these panels are listed in Fig 1A. (A, C, and D) The numerical data is listed in S3 Data. hESC, human embryonic stem cell; ICM, inner cell mass; scRNA-seq, single-cell RNA sequencing; SEM, standard error of the mean; TE, trophectoderm; 5fCpG, 5-formylcytosine phosphate guanine.
(TIF)

S3 Fig The 5fCpG dynamic in genomic regions during human early embryonic development. (A) The line chart showing the 5fCpG level in distinct genomic regions of human early embryo and hESC. The center is the mean of 5fCpG level, and error bars are SEM. (B) The line chart showing the 5fCpG level in three types of promoter, HCP (left panel), ICP (middle panel), and LCP (right panel) according to the CpG density. The center is the mean of 5fCpG level, and error bars are SEM. (A–B) The numerical data are listed in S3 Data. (C) Box plot showing the variance of 5fCpG abundance during human early embryonic development. The variance was calculated in 1-kb window. The box indicates the median, 25% quartile, and 75% quartile. The whisker represents the 1.5 times of IQR. The numerical data are listed in S4 Data. (A–C) The sample size in these panels are listed in Fig 1A. (D) t-SNE analysis of 5fCpG-marked windows at different window sizes. CpG, cytosine phosphate guanine; HCP, high-density CpG promoter; hESC, human embryonic stem cell; ICP, intermediated-density CpG promoter; IQR, interquartile range; LCP, low-density CpG promoter; SEM, standard error of the mean; t-SNE, t-distributed stochastic neighbor embedding; 5fCpG, 5-formylcytosine phosphate guanine.
(TIF)

S4 Fig PCA of 5fCpG-marked genomic windows at the windows size of 1 kb, 10 kb, 100 kb, 1 Mb, 10 Mb. PCA, principal component analysis; 5fCpG, 5-formylcytosine phosphate guanine.
(TIF)

S5 Fig 5fCpG distribution and enrichment on repeat elements. (A) Enrichment analysis of 5fCpG site on different repeat elements. (B) The line chart showing the 5fCpG level on distinct repeat elements. The center is the mean of 5fCpG level and error bars are SEM. (C) The line char showing the expression level of L1 (left panel) and ERVK (right panel) with 5fCpG modification or randomly seletcted ones only with unmodified CpG. The significance based on Student t-test is denoted. The scRNA-seq data of human early embryos are from GSE36552. (D) The line char showing the DNA methylation levels of L1 (left panel) and ERVK (right panel) with 5fCpG modification or randomly seletcted ones only with unmodified CpG. The significance based on Student *t* test is denoted.The DNA methylaiton data of human early embryos are from GSE81233. (E) Enrichment analysis of gamete-specific, gamete-shared, pronuclei-specific, and pronuclei-shared 5fCpG-marked regions (1-kb window) in repeat elements. (A–E) The sample size in these panels are listed in Fig 1A, and the numerical data are listed in S3 Data. CpG, cytosine phosphate guanine; ERVK, endogenous retrovirus-K; SEM, standard error of the mean; scRNA-seq, single-cell RNA sequencing; 5fCpG, 5-formylcytosine phosphate guanine.
(TIF)

S6 Fig The 5fCpG level in blastomeres of paired two-cell. (A) The 5fCpG level in blastomeres of paired two-cell in whole genome (*n* = 4). The dots in red and blue represent the mean of 5fCpG level in two blastomeres, respectively. (B) The line chart showing the 5fCpG level of paired blastomeres in two-cell stage in different genomic regions (*n* = 4). The dots represent the mean of 5fCpG level. (C) The line chart showing the 5fCpG level of paired blastomeres in two-cell stage in different repeat elements and the subfamilies of them (*n* = 4). The dots represent the mean of 5fCpG level. (A–C) The numerical data is listed in S3 Data. 5fCpG, 5-formylcytosine phosphate guanine.
(TIF)

S1 Table Basic summary of human preimplantation embryo sample information. (XLSX)

S2 Table The sequence of model DNA. (XLSX)

S1 Data The individual numerical values in Fig 2A and 2B, Fig 2D and 2E, Fig 3A and 3B, Fig 4E and 4F, Fig 5B–5D, Fig 6B–6D. (XLS)

S2 Data The individual numerical values in Fig 2F. (ZIP)

S3 Data The individual numerical values in S2A Fig, S2C and S2D Fig, S3A and S3B Fig, S5A–S5E Fig, S6A–S6C Fig. (XLS)

S4 Data The individual numerical values in S3C Fig. (ZIP)

## Abbreviations

bp: base pairs
CGI: CpG island
ChIP-seq: chromatin immunoprecipitation sequencing
CLEVER-seq: chemical-labeling-enabled C-to-T conversion sequencing
CNV: copy number variation
CpG: cytosine phosphate guanine
CTCF: CCCTC-binding factor
dbSNP: database of single nucleotide polymorphism
DHS: DNase I hypersensitive sites
ERVK: endogenous retrovirus-K
GEO: Gene Expression Omnibus
HB: Holm–Bonferroni
HCP: high-density CpG promoter
hESC: human embryonic stem cell
ICM: inner cell mass
ICP: intermediated-density CpG promoter
ICSI: intracytoplasmic sperm injection
IVF: in vitro fertilisation
LCP: low-density CpG promoter
LINE: long interspersed nuclear element
LTR: long terminal repeat
MALBAC: multiple annealing- and looping-based amplification cycles
MIR: mammalian-wide interspersed repeat
NDR: nucleosome-depleted region
PCA: principal component analysis
PCR: polymerase chain reaction
RPKM: reads per kilobase of transcript per million mapped reads
scCOOL-seq: chromatin overall omic-scale landscape sequencing
SINE: short interspersed nuclear element
SNP: single nucleotide polymorphism
SVA: SINE/variable number of tandem repeats/Alu
TE: trophectoderm
TET: ten-eleven translocation protein family
t-SNE: t-distributed stochastic neighbor embedding
TTS: transcriptional termination site
UTR: untranslated region
5caC: 5-carboxylcytosine
5fC: 5-formylcytosine
5fCpG: 5-formylcytosine phosphate guanine
5hmC: 5-hydroxymethylcytosine
5mC: 5-methylcytosine
