# Supplementary figures and images for: 5-Formylcytosine landscapes of human preimplantation embryos at single-cell resolution
Source: PLoS Biol. 2020 Jul 30;18(7):e3000799. doi: 10.1371/journal.pbio.3000799 (PMC7419013; doi:10.1371/journal.pbio.3000799)

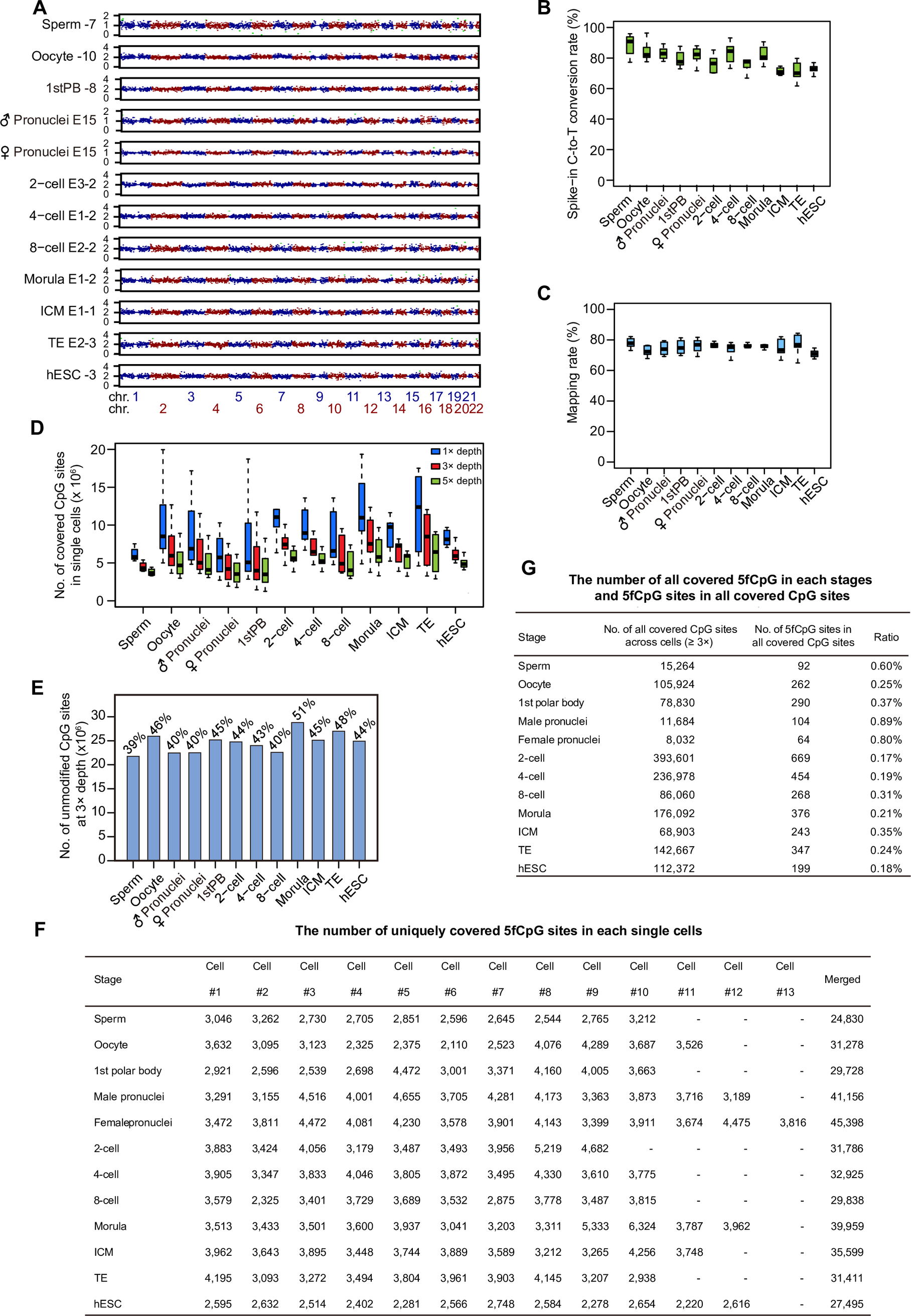

Supplement: S1 Fig — (A) Representative copy number variation plot showing that only euploid cell were retained for further analysis in this study. (B) Box plot showing the C-to-T conversion rate of spike-in oligo DNA which contained a 5fC modification in each stages. The box indicates the median, 25% quartile, and 75% quartile. The whisker represents the 1.5 times of IQR. (C) Box plot showing the mapping rate of 130 libraries in each stages. The box indicates the median, 25% quartile, and 75% quartile. The whisker represents the 1.5 times of IQR. (D) Box plot showing the CpG coverage at 1× (blue), 3× (red), 5× (green) depth of libraries in different stages. The box indicates the median, 25% quartile, and 75% quartile. The whisker represents the 1.5 times of IQR. (E) Histogram showing the number of covered unmodified CpG sites in each stages at 3× depth. (B–E) The sample size in these panels are listed in Fig 1A; the numerical data are listed in S1 Table. (F) The table showing the number of 5fCpG sites identified in each single cell and the number of stage-merged 5fCpG sites. (G) The table summarizing the number of CpG sites covered by all cells in each stage at 3× depth, the number as well as the ratio of 5fCpG sites in all covered CpG sites. CLEVER-seq, chemical-labeling-enabled C-to-T conversion sequencing; CpG, chemical-labeling-enabled C-to-T conversion sequencing; IQR, interquartile range; 5fC, 5-formylcytosine; 5fCpG, 5-formylcytosine phosphate guanine. (TIF) [file pbio.3000799.s001.tif]

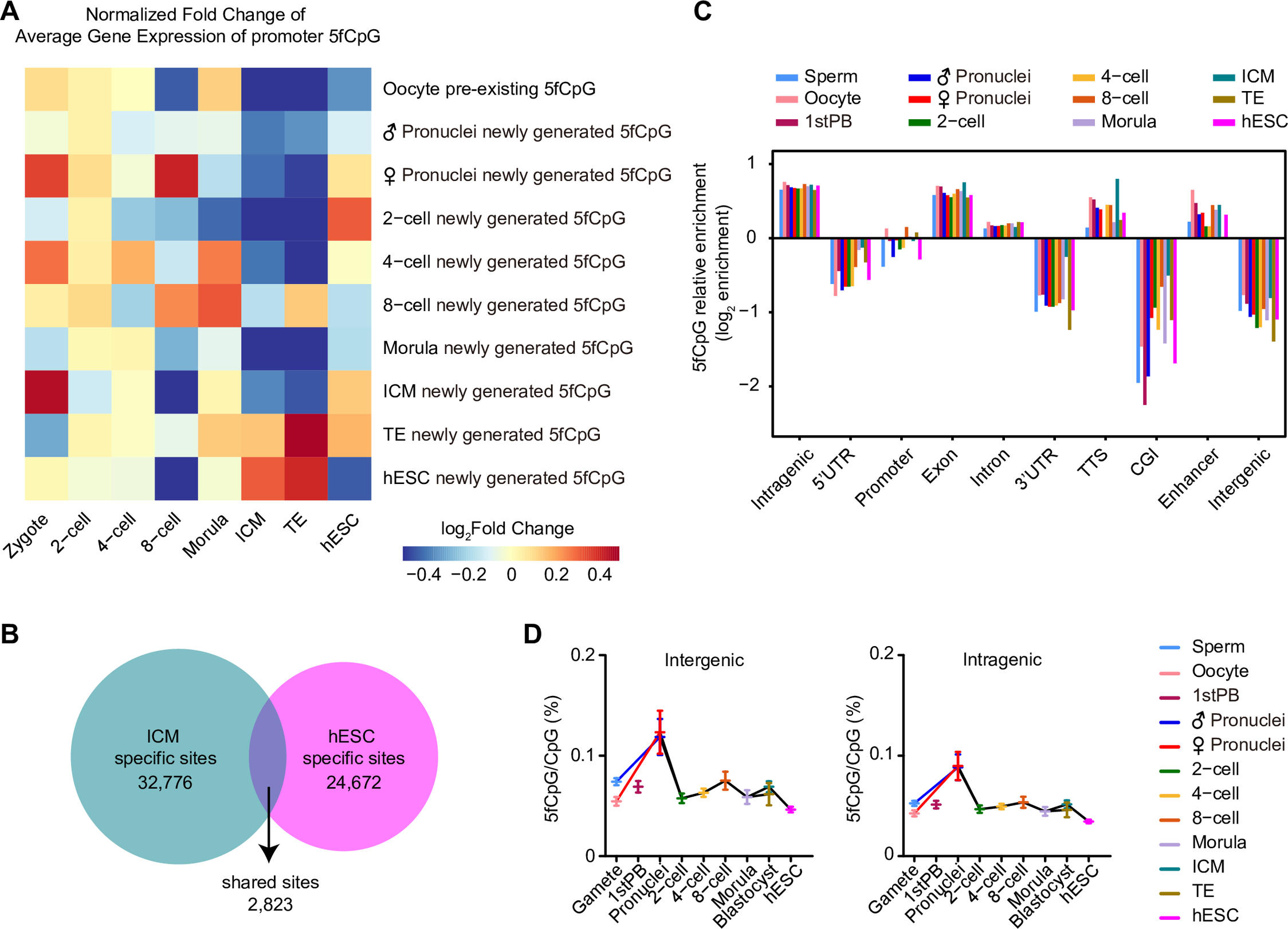

Supplement: S2 Fig — (A) The normalized fold changes of average gene expression levels between two consecutive stages for genes with 5fCpG in promoters (TSS ± 2 kb). The hESC was compared with ICM, whereas both ICM and TE were compared with morula.The color from blue to red indicate the values of log2 of expression level fold change between two consecutive stages from low to high. The scRNA-seq data of human early embryos are from GSE36552. (B) Venn diagram showing the 5fCpG site overlap between ICM and hESC. The number of ICM-specific, hESC-specific, and their shared 5fCpG sites are listed in the diagram. (C) Bar plot showing the relative enrichment of 5fCpG sites on different genomic region. (D) Line chart showing the 5fCpG level in intergenic and intragenic region across human early embryo developmental stages. The center is the mean of 5fCpG level and error bars are SEM. (A–D) The sample size in these panels are listed in Fig 1A. (A, C, and D) The numerical data is listed in S3 Data. hESC, human embryonic stem cell; ICM, inner cell mass; scRNA-seq, single-cell RNA sequencing; SEM, standard error of the mean; TE, trophectoderm; 5fCpG, 5-formylcytosine phosphate guanine. (TIF) [file pbio.3000799.s002.tif]

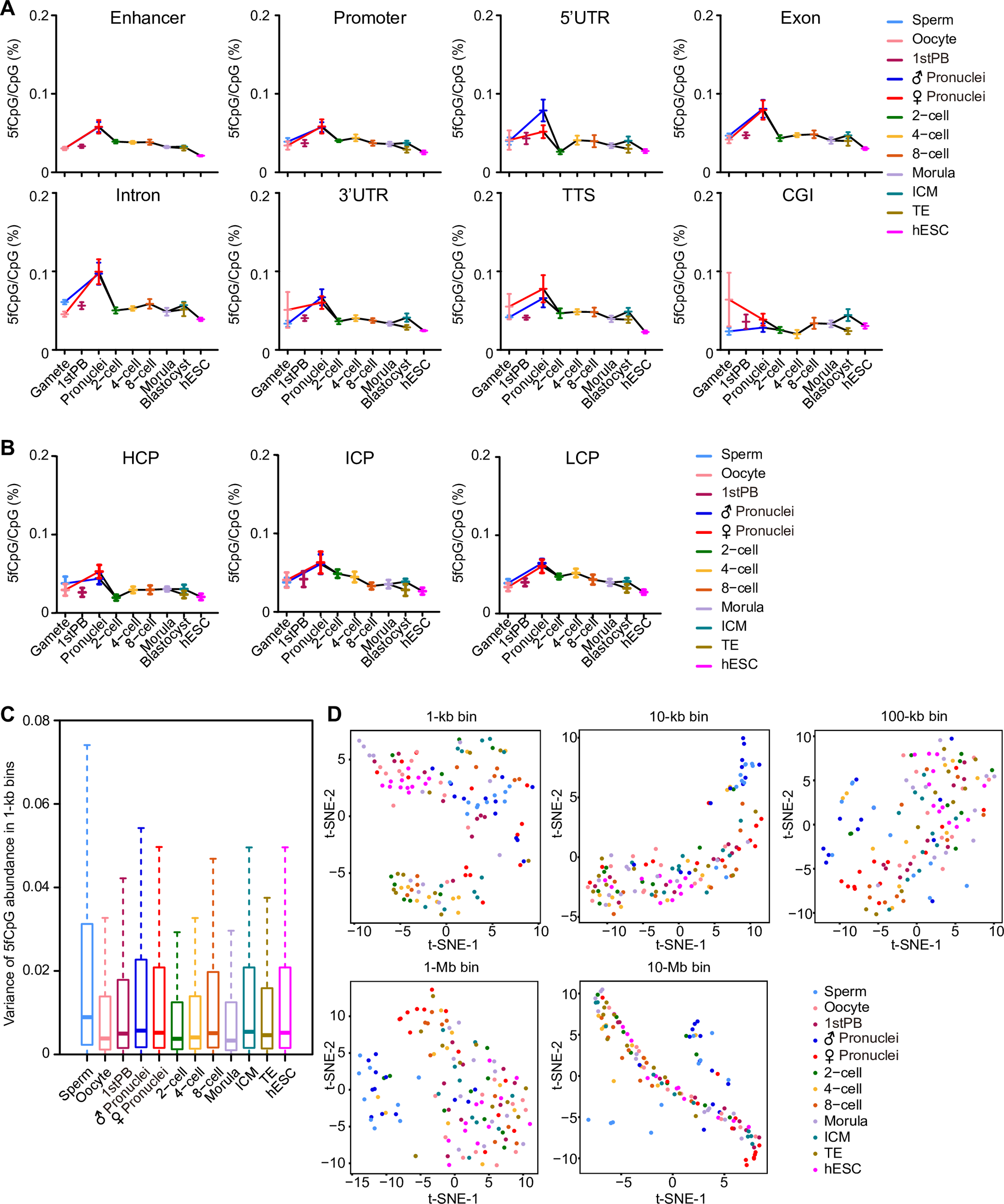

Supplement: S3 Fig — (A) The line chart showing the 5fCpG level in distinct genomic regions of human early embryo and hESC. The center is the mean of 5fCpG level, and error bars are SEM. (B) The line chart showing the 5fCpG level in three types of promoter, HCP (left panel), ICP (middle panel), and LCP (right panel) according to the CpG density. The center is the mean of 5fCpG level, and error bars are SEM. (A–B) The numerical data are listed in S3 Data. (C) Box plot showing the variance of 5fCpG abundance during human early embryonic development. The variance was calculated in 1-kb window. The box indicates the median, 25% quartile, and 75% quartile. The whisker represents the 1.5 times of IQR. The numerical data are listed in S4 Data. (A–C) The sample size in these panels are listed in Fig 1A. (D) t-SNE analysis of 5fCpG-marked windows at different window sizes. CpG, cytosine phosphate guanine; HCP, high-density CpG promoter; hESC, human embryonic stem cell; ICP, intermediated-density CpG promoter; IQR, interquartile range; LCP, low-density CpG promoter; SEM, standard error of the mean; t-SNE, t-distributed stochastic neighbor embedding; 5fCpG, 5-formylcytosine phosphate guanine. (TIF) [file pbio.3000799.s003.tif]

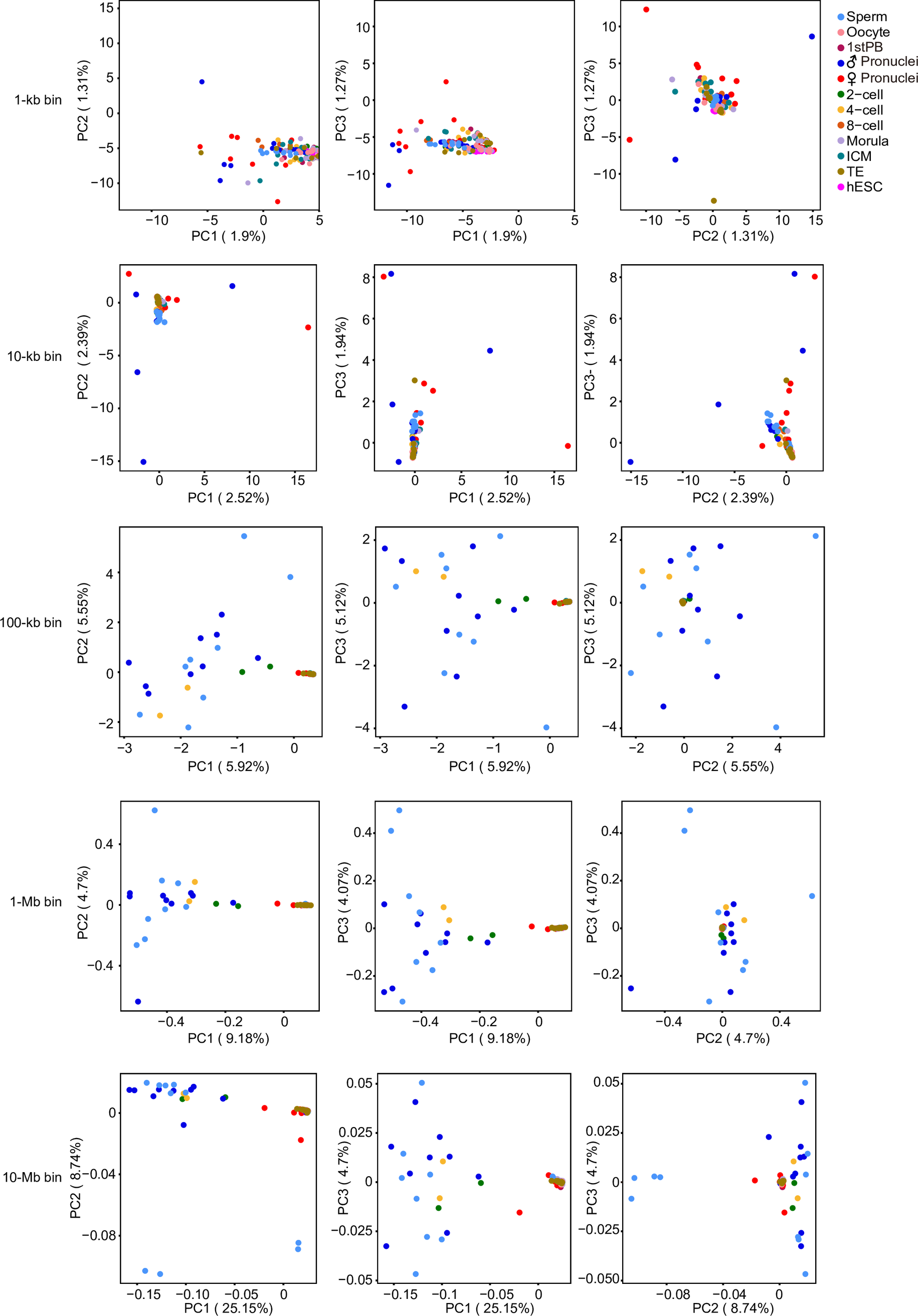

Supplement: S4 Fig — PCA, principal component analysis; 5fCpG, 5-formylcytosine phosphate guanine. (TIF) [file pbio.3000799.s004.tif]

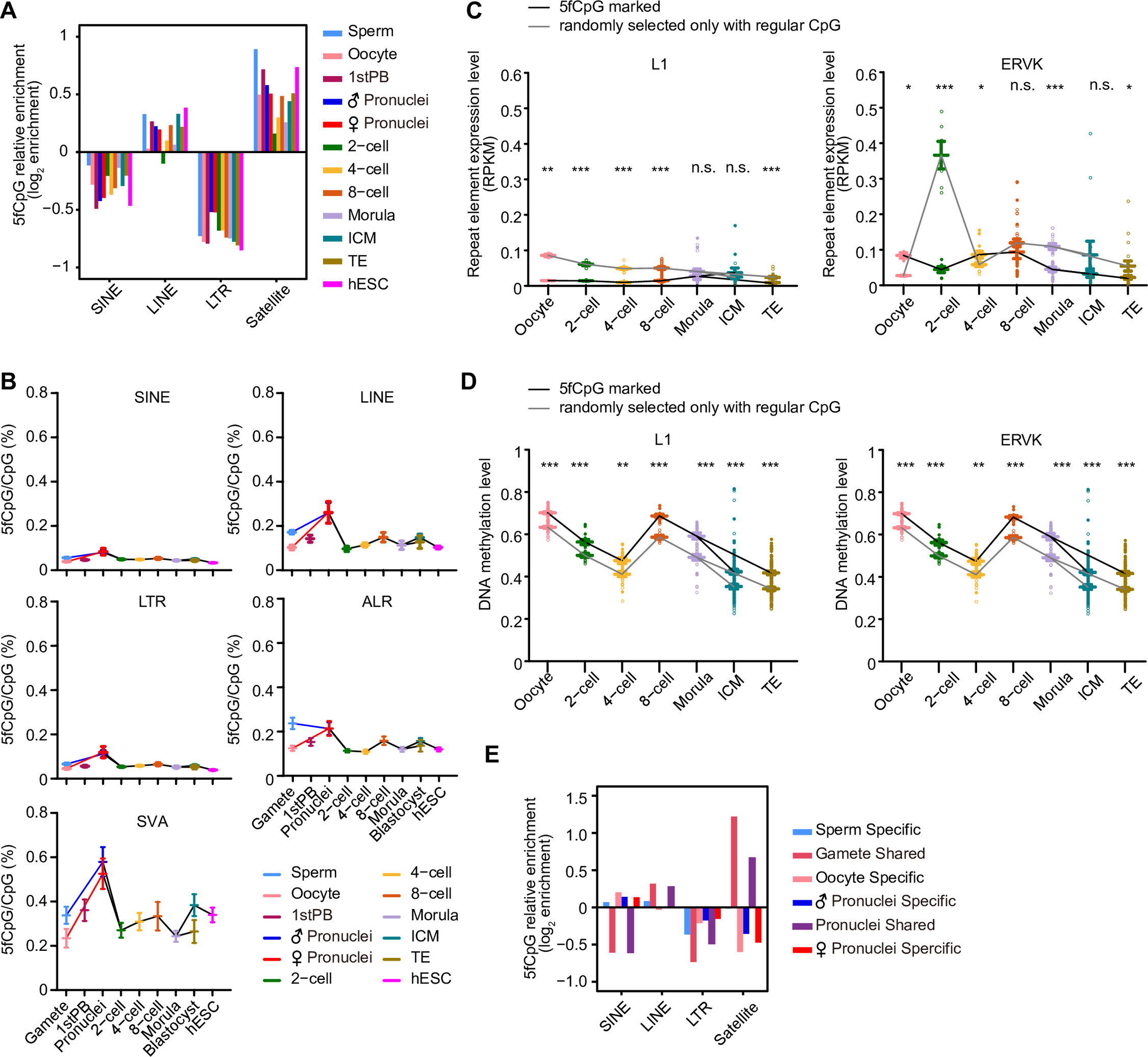

Supplement: S5 Fig — (A) Enrichment analysis of 5fCpG site on different repeat elements. (B) The line chart showing the 5fCpG level on distinct repeat elements. The center is the mean of 5fCpG level and error bars are SEM. (C) The line char showing the expression level of L1 (left panel) and ERVK (right panel) with 5fCpG modification or randomly seletcted ones only with unmodified CpG. The significance based on Student t-test is denoted. The scRNA-seq data of human early embryos are from GSE36552. (D) The line char showing the DNA methylation levels of L1 (left panel) and ERVK (right panel) with 5fCpG modification or randomly seletcted ones only with unmodified CpG. The significance based on Student t test is denoted.The DNA methylaiton data of human early embryos are from GSE81233. (E) Enrichment analysis of gamete-specific, gamete-shared, pronuclei-specific, and pronuclei-shared 5fCpG-marked regions (1-kb window) in repeat elements. (A–E) The sample size in these panels are listed in Fig 1A, and the numerical data are listed in S3 Data. CpG, cytosine phosphate guanine; ERVK, endogenous retrovirus-K; SEM, standard error of the mean; scRNA-seq, single-cell RNA sequencing; 5fCpG, 5-formylcytosine phosphate guanine. (TIF) [file pbio.3000799.s005.tif]

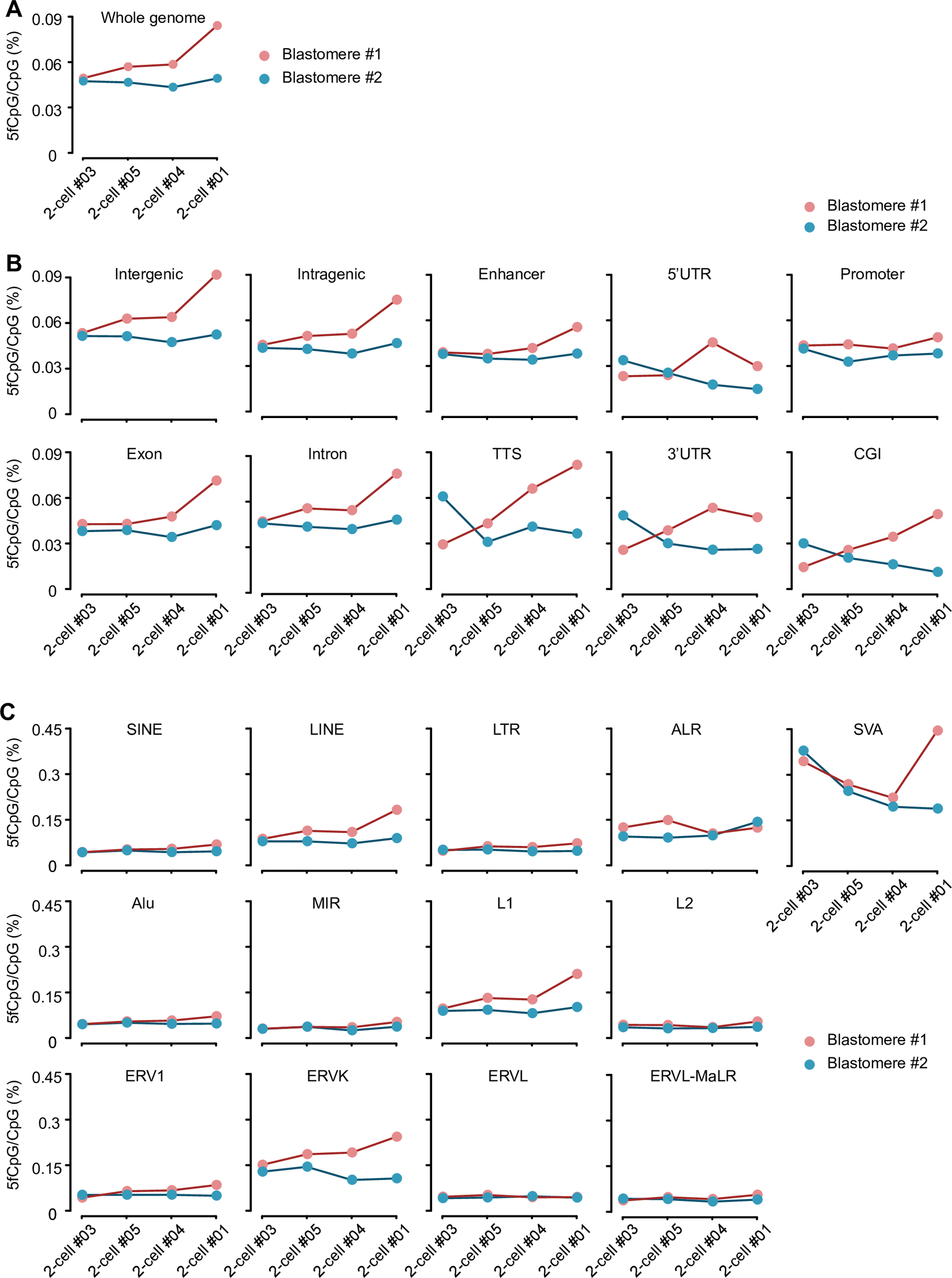

Supplement: S6 Fig — (A) The 5fCpG level in blastomeres of paired two-cell in whole genome (n = 4). The dots in red and blue represent the mean of 5fCpG level in two blastomeres, respectively. (B) The line chart showing the 5fCpG level of paired blastomeres in two-cell stage in different genomic regions (n = 4). The dots represent the mean of 5fCpG level. (C) The line chart showing the 5fCpG level of paired blastomeres in two-cell stage in different repeat elements and the subfamilies of them (n = 4). The dots represent the mean of 5fCpG level. (A–C) The numerical data is listed in S3 Data. 5fCpG, 5-formylcytosine phosphate guanine. (TIF) [file pbio.3000799.s006.tif]
